# Public health and criminal justice funding for firearm injury prevention research in the United States

**DOI:** 10.1186/s40621-025-00644-3

**Published:** 2026-02-02

**Authors:** John C. Lin, Christopher Chang, Madison S. McCarthy, Lily N. Tran, Chaerim Kang, Abe Baker-Butler, Lauren A. Magee, Guangyu Tong, Megan L. Ranney

**Affiliations:** 1 Department of Medicine, Perelman School of Medicine, University of Pennsylvania, Philadelphia, PA USA; 2 The Warren Alpert Medical School, Brown University, Providence, RI USA; 3 Department of Politics and International Studies, University of Cambridge, Cambridge, UK; 4 Yale School of Public Health, 60 College St, New Haven, CT 06510 USA; 5 Division of Biology and Medicine, Brown University, Providence, RI USA; 6 The Warren Alpert Medical School, Brown University, Providence, RI USA; 7 Department of Pediatrics, Indiana University School of Medicine, Indianapolis, IN USA; 8 Department of Emergency Medicine, Yale School of Medicine, New Haven, CT USA

**Keywords:** Firearm injury prevention research, Trends in federal funding, Public health, Criminal justice

## Abstract

**Objectives:**

To compare the differences in US federal funding sources for firearm injury prevention and publications.

**Methods:**

We extracted publications from three literature databases and grant data for five federal agencies (CDC, NIH, SAMHSA, NSF, NIJ) through federal archives from 2020 to 2022, excluding case studies, editorials, and literature reviews. Specific funding sources for publications were further extracted. We calculated funding-to-publication (F-P) ratios for federal public health, science, and criminal justice agencies and tested associations. Health agencies with grant data included the CDC, NIH, and SAMHSA and were categorized as public health agencies. The NSF was classified as science and engineering. The NIJ was classified as criminal justice.

**Results:**

The three largest funders were the NIH, CDC, and NIJ, and were associated with the most publications, with health agencies funding most studies (86%). Health agencies had higher F-P ratios than the NSF and NIJ. Public health funders were more likely to fund experimental studies, studies related to suicide, and unintentional firearm injury prevention. NIJ and NSF were more likely to support research on mass shootings. Most studies funded by all agencies examined community violence.

**Conclusions:**

The NIH and CDC supported most firearm-injury-related grant funding and subsequent publications from 2020 to 2022. Differences in funding existed depending on the types of firearm injury. Federal funding is imperative to advance the science of firearm injury prevention, and future funding across federal agencies should be aligned with national public health and safety needs.

**Supplementary Information:**

The online version contains supplementary material available at 10.1186/s40621-025-00644-3.

## Introduction

Firearm violence has killed more than 40,000 Americans every year, with approximately 85,000 more who are shot and wounded. The repercussions of such violence are often extensive, spanning beyond the physical, into the psychological, social, and economic domains [1]. The US Congress passed the Dickey Amendment in 1996, effectively preventing public health (PH) agencies such as the Centers for Disease Control and Prevention (CDC) and later the National Institutes of Health (NIH) from funding firearm injury prevention research [2, 3]. Despite the passing of the Dickey Amendment, the National Institute of Justice (NIJ) continued funding research on criminal justice (CJ) approaches to firearm injury, while other agencies informally supported firearm injury prevention research [2, 4]. 

PH and CJ research approaches often differ. CJ primarily focuses on deterrence-based interventions and criminal investigations, whereas PH often focuses on prevention through social-ecological factors [5]. These differences may be reflected in the types of firearm injury prevention research funded, publications, and which evidence-based policies are implemented to reduce firearm injury. For example, public health’s “upstream approach” to community-based violence prevention encompasses interventions addressing environmental improvements, [6, 7] services to support social and economic needs, [8] and clinical links that connect healthcare providers with community-based resources and violence intervention services, [9, 10] whereas criminal justice tends to focus on policing-related interventions [11]. 

Amid recent reductions in federal appropriations for scientific research, concerns have grown over the long-term capacity of public institutions to sustain critical areas of inquiry, including firearm injury prevention. Although the CDC and NIH resumed limited funding for firearm injury research in 2019 after a two-decade hiatus, this support remains precarious. Recent proposals to eliminate such funding entirely highlight the ongoing politicization of the field and underscore the need to better understand how federal agencies have supported this research historically and structurally [12]. Thus, in this study, we compared firearm injury prevention research funding and their resulting publications by US federal agencies to explore the priorities, gaps, and methodological orientations that shape the national investment in research to prevent firearm injuries.

## Methods

Our search strategies have been described elsewhere [2]. 

We developed our methods (Table S1) with an information methodologist. In April 2023, we extracted publications from three literature databases and grant data for five federal agencies through federal archives from 2020 to 2022 after Congress began providing funding for firearm injury prevention research (Table 1). We defined firearm injury prevention research broadly, encompassing experimental or epidemiologic investigations. We excluded case studies, editorials, and literature reviews. Four investigators (JCL/CC/MSM/ABB) reviewed full texts, with the senior investigator mediating disagreements, then extracted specific funding sources for publications (JCL/CC/LNT/CK).

We conducted analyses in Stata Version 18.0 (StataCorp, College Station, TX). We described the funding ratio to publication (F-P ratio) for PH, science, and CJ funding agencies, and tested associations with study characteristics using Fisher’s exact tests. Health agencies with grant data included the CDC, NIH, and Substance Abuse and Mental Health Services Administration (SAMHSA). The NSF was classified as science and engineering as it primarily funds research in those fields; however, it also funds interdisciplinary research. The NIJ was classified as criminal justice.

## Results

Our study found a mean of 65.1 publications per year and $536,600 in grant funding across all agencies for the fiscal years 2020–2022. The breakdown, by funder, are also reported (Table 1). The three largest funders were the NIH, CDC, and NIJ; they were associated with the most publications. Health agencies funded most studies (86% [*n* = 470/548]) and had higher F-P ratios than the NSF and NIJ.

Publications were further characterized based on funding sources (Table 2). PH funders were more likely to fund experimental studies and less likely to fund quasi-experimental studies than the NIJ (*p* = 0.0178). PH agencies were more likely to fund studies related to suicide (*p* < 0.0001) and unintentional firearm injury prevention (*p* = 0.0013); NIJ and NSF were more likely to support research on mass shootings (*p* < 0.001). Most studies funded by all agencies (PH: 61% [*n* = 285]), NSF: 78% [*n* = 14]), and NIJ: 65% [*n* = 39]) examined community violence.

**Table 1:** Mean numbers of firearm injury prevention research publications and total grant funding (in $100K) by funding agency per year, 2020–2022

Funding agency^a, b^	Agency type	No. publications (*P*)	Mean annual grant funding (F), $100K	F-*P* ($100K) Ratio,
National Institutes of Health (NIH)	Health	41.3	335.0	8.1
Centers for Disease Control and Prevention (CDC)	Health	13	163.0	12.5
Substance Abuse and Mental Health Services Administration (SAMHSA)	Health	1	8.0	8.0
National Science Foundation (NSF)	Science	2.5	10	4.0
National Institute of Justice (NIJ)	Criminal Justice	7.3	20.6	2.8

^a^ Publications acknowledging multiple funding agencies or sources were counted toward each funder

^b^ Analyses were limited to 2020–2022 to evaluate the initial effects of renewed federal appropriations, with additional analyses spanning 1985–2022 to contextualize these findings across pre-, restricted-, and post-Dickey Amendment funding periods

**Table 2:** Association of Characteristics, study Topics, and funding agency for federally funded Publications^a^

Study characteristics	Health agencies (*n* = 526)	Science agencies (*n* = 18)	Criminal justice agencies (*n* = 60)	*P*-values^c, d^
Data Source Year, n(%)				
1985–1996	38 (7.2)	0 (0.0)	1 (1.7)	
1997–2013	145 (27.6)	4 (22.2)	20 (33.3)	0.2747
2014–2019	154 (29.3)	9 (50.0)	17 (28.3)	
2020–2022	189 (35.9)	5 (27.8)	22 (36.7)	
Study Design, n(%)				
Experimental	27 (5.1)	1 (5.6)	0 (0.0)	
Observational	440 (83.7)	12 (66.7)	48 (80.0)	**0.0170**
Qualitative	22 (4.2)	1 (5.6)	2 (3.3)	
Quasi-Experimental	37 (7.0)	4 (22.2)	10 (16.7)	
Study Population, n(%)				
City/County	163 (31.0)	11(61.1)	22 (36.7)	
Military	8 (1.5)	0 (0.0)	0 (0.0)	0.1277
National	253 (48.1)	7 (38.9)	26 (43.3)	
State	96 (18.3)	0 (0.0)	12 (20.0)	
Missing	6 (1.1)	0 (0.0)	0 (0.0)	
Population Age, n(%)				
Adult	118 (22.4)	2 (11.1)	9 (15.0)	
Pediatric (up to 21)	175 (33.3)	4 (22.2)	12 (20.0)	
Both	177 (33.7)	7 (38.9)	23 (38.3)	0.3303
Missing	56 (10.6)	5 (27.8)	16 (26.7)	
Type of Injury^b^, n(%)				
Community violence	285 (60.6)	14 (77.8)	39 (65.0)	0.3134
Suicide	269 (57.2)	6 (33.3)	4 (6.7)	**< 0.0001**
Domestic violence	26 (5.5)	0 (0.0)	3 (5.0)	0.9070
Mass shooting	5 (1.1)	3 (16.7)	4 (6.7)	**0.0002**
Unintentional	106 (22.6)	2 (11.1)	3 (5.0)	**0.0013**
Other injury	101 (21.5)	3 (16.7)	3 (5.0)	**0.0035**

Statistically significant relationships (*p* < 0.05) were bolded

^a^ Funding agencies (columns) are not mutually exclusive. 22 out of 526 publications acknowledged agencies from two categories

^b^ Each study could investigate multiple outcomes. Testing for differences was performed for each row

^c^ P values were based on Fisher’s exact tests, excluding missing data

^d^ Analyses were limited to 2020–2022 to evaluate the initial effects of renewed federal appropriations, with additional analyses spanning 1985–2022 to contextualize these findings across pre-, restricted-, and post-Dickey Amendment funding periods

## Discussion

The NIH and CDC supported most firearm-injury-related grant funding and subsequent publications in 2020–2022. Funding per publication was higher for PH agencies, possibly due to more funding allocated towards experimental studies (which are more expensive) [13]. F-P ratios help identify structural differences in how agencies support firearm injury prevention research and may serve as a metric to evaluate how efficiently funds are used for evidence-based research. Higher ratios among public health agencies (e.g., NIH, CDC) likely reflect the greater costs associated with experimental and multi-site studies, whereas lower ratios within criminal justice or science agencies (e.g., NIJ, NSF) may indicate smaller or less resource-intensive projects.

Differences in funding existed according to the types of firearm injury. Community violence—the leading type of non-fatal firearm injury—predominated for all agencies. A melding of CJ and PH approaches to all types of firearm injury may result in new prevention strategies [14]. 

Limitations include small sample sizes, missed publications and grants, and possible miscategorization of research. Furthermore, publication processes, expectations, and research goals may also differ by field and by agency. The timing of the distribution of funds as well as when a project was initiated could only been obtained by publicly available data and may not reflect funding trends during the same period. Lastly, while other criminal justice grants were made during this period through agencies such as the OOJDP, BJA, and BJS, we excluded these as the present analysis focused primarily on the NIJ as the DOJ’s main research agency.

Historically, firearm injury prevention research was underfunded by federal agencies relative to other leading causes of death due to interpretations of the Dickey Amendment [15]. Federal funding is imperative to advance the science of firearm injury prevention, and future funding across federal agencies should be aligned with national public health and safety needs.

## Supplementary Information

Supplementary Material 1

## Data Availability

No datasets were generated or analysed during the current study.

## Abbreviations

PH: Public health
CDC: Centers for Disease Control and Prevention
NIH: National Institutes of Health
NIJ: National Institute of Justice
CJ: Criminal justice
F-P ratio: Unding ratio to publication
SAMHSA: Substance Abuse and Mental Health Services Administration
