# Supplementary material for: Public health and criminal justice funding for firearm injury prevention research in the United States
Source: Inj Epidemiol. 2026 Feb 2;13:10. doi: 10.1186/s40621-025-00644-3 (PMC12866236; doi:10.1186/s40621-025-00644-3)
Supplement: Supplementary file 1 — Supplementary Material 1 [file 40621_2025_644_MOESM1_ESM.docx]

**Table S1: Search terms by grant database**

| **Database** | **Agencies** | **Terms** |
| --- | --- | --- |
| NIH RePORTER | AHRQ (1993-2023), ACF (2013-2017), (1993-1997), CDC (1993-2023), FDA (1993-2023), NIH (1985-2023), and VA (2009-2023) | (firearm OR handgun OR gun) NOT (microscopy OR genomic OR gene OR assay OR ion OR microbiome OR proteomic OR mass OR spec OR sequence OR microbiota OR micro* OR biomarker OR cancer OR biolog* OR imaging OR Vitro OR Homeostasis OR XRAY OR protein OR Virus OR sequencing) |
| NIJ Listing of Funded Projects | NIJ (2015-2022) | Firearm or Firearms or Gun or Guns or Handgun or Handguns or Weapon or Weapons or Gunshot or Gunshots |
| NSF Award Search | NSF (1959-2023) | Firearm or Firearms or Gun or Guns or Handgun or Handguns |
| SAMHSA Grants Dashboard | SAMHSA (2014-2023) | Firearm or Firearms or Gun or Guns or Handgun or Handguns |

ACF, Administration for Children and Families; AHRQ, Agency for Healthcare Research and Quality; CDC, Centers for Disease Control and Prevention; FDA, Food and Drug Administration; HRSA, Health Resources and Services Administration; NIH, National Institutes of Health; NIJ, National Institute of Justice; NSF, National Science Foundation; SAMHSA, Substance Abuse and Mental Health Services Administration; TAGGS, Tracking Accountability in Government Grants System; VA, Veterans Affairs.

We extracted federal grant data from the earliest year available up to 2023, if possible. VA data was available beginning in 2009, ACF data in 2013, SAMHSA in 2014, and NIJ in 2015.
